# Response to Zhou and Robinson

**DOI:** 10.1186/s13059-015-0782-2

**Published:** 2015-10-08

**Authors:** Doron Betel, Nicholas D. Socci, Raya Khanin, Christopher E. Mason, Franck Rapaport

**Affiliations:** Weill Cornell Medical College, 1305 York Avenue, New York, NY 10021 USA; Memorial Sloan Kettering Cancer Center, 1275 York Avenue, New York, NY 10065 USA

The choice of algorithmic tool for analysis of genomic data is often guided by personal preference and convenience rather than clear scientific criteria. In a recent paper (Rapaport *et al*. Genome Biol. 2013;14:R95) we performed an objective analysis of widely used RNA-seq differential expression (DE) programs in order to understand the performance of the various tools with respect to different evaluation measures. One such measure was the interpretation of DE when there is no detectable signal from one of the contrasting conditions. Zhou and Robinson present insightful reanalysis of this comparison. They present two claims. The first is that there was a coding error when calculating the normalized edgeR count values. Second, that our use of signal-to-noise ratio (S/N) as a measure of sensitivity and specificity (in the form of ROC) is incorrect. This is because the mean and variance are correlated in count data and therefore the signal (mean) and noise (standard deviation) are not two independent measures. Perhaps, more importantly, the ROC analysis was not based on the same set of truth set for all methods since the same S/N threshold value corresponds to very different genes sets for each method.

The authors are correct in both these points and we appreciate the effort invested in bringing those to light. Our coding error was omitting scaling by library size when calculating the normalized edgeR values. However, this error has very little impact on the analysis as presented in Fig. 4 of our manuscript and does not change its conclusions(see erratum to the manuscript).

The most salient point raised is the use of the same S/N threshold in the ROC evaluation. The main argument is that the scale of mean and variance is different between the different methods and therefore the methods are not evaluating the same sets of genes. That is correct and we agree with these points. The second point raised is that since the mean and variance are highly correlated in count data calculating S/N using transformed mean variance values is more appropriate (Fig. 2 in Zhou and Robinson’s response). It is notable that the ROC performances of edgeR and DESeq2 relative to limma in Fig. 2d are similar to those presented in Fig. 4b of Rapaport *et al*. suggesting that selecting a fixed set of labels is not the major reason for the differences in performances. The improvement in the ROC performance for edgeR (and DESeq2) relative to limma is attributed to the transformation of the mean variance values (Fig. 2b and c).

The ROC analysis is only one part of much broader analyses of this subset of genes. Our analysis was originally motivated by the observation that often genes with similar mean expression values, in the condition in which they are expressed, had different *P* values. This was contrary to our initial expectation, since these genes had similar mean expression values and therefore similar log expression ratios we anticipated similar *P* values. Moreover, we noticed that some genes with higher expression values had less significant *P* values than genes with lower expression values (and similar standard deviation). This introduces a problem when selecting genes for subsequent expression validation and functional importance. We were interested in characterizing this further and gain a better understanding of this discrepancy. As noted in both Rapaport *et al*. and in the response from Zhou and Robinson, most of these genes are clearly differentially expressed and are correctly detected as such by all methods. Hence the challenge is not in correct identification of DE (that is, rejecting the null hypothesis) but rather meaningful ordering of the DE genes. Therefore, ranking this specific set of genes by their *P* values of differential expression indicates the confidence in experimental measurement rather than rejection of the null hypothesis (which is trivial in this case since there is a clear change in expression). In our paper (Fig. 4a) we show that limma and PoissonSeq packages have a clear monotonic relationship between the S/N and *P* value assigned to difference in expression for this particular subset of genes with zero value in one condition. In contrast, DESeq, edgeR, and cuffdiff did not display this monotonic relationship. It would be interesting to see the impact of mean variance scaling on this correlation.

